# Proton ARC based LATTICE radiation therapy: feasibility study, energy layer optimization and LET optimization

**DOI:** 10.1088/1361-6560/ad8855

**Published:** 2024-10-25

**Authors:** Ya-Nan Zhu, Weijie Zhang, Jufri Setianegara, Yuting Lin, Erik Traneus, Yong Long, Xiaoqun Zhang, Rajeev Badkul, David Akhavan, Fen Wang, Ronald C Chen, Hao Gao

**Affiliations:** 1 Department of Radiation Oncology, University of Kansas Medical Center, Kansas, United States of America; 2 RaySearch Laboratories AB, Stockholm, Sweden; 3 University of Michigan-Shanghai Jiao Tong University Joint Institute, Shanghai Jiao Tong University, Shanghai, People’s Republic of China; 4 Institute of Natural Sciences and School of Mathematics, Shanghai Jiao Tong University, Shanghai, People’s Republic of China

**Keywords:** SFRT, LATTICE, proton ARC, energy layer optimization

## Abstract

**Objective.:**

LATTICE, a spatially fractionated radiation therapy (SFRT) modality, is a 3D generalization of GRID and delivers highly modulated peak-valley spatial dose distribution to tumor targets, characterized by peak-to-valley dose ratio (PVDR). Proton LATTICE is highly desirable, because of the potential synergy of the benefit from protons compared to photons, and the benefit from LATTICE compared to GRID. Proton LATTICE using standard proton RT via intensity modulated proton therapy (IMPT) (with a few beam angles) can be problematic with poor target dose coverage and high dose spill to organs-at-risk (OAR). This work will develop novel proton LATTICE method via proton ARC (with many beam angles) to overcome these challenges in target coverage and OAR sparing, with optimized delivery efficiency via energy layer optimization and optimized biological dose distribution via linear energy transfer (LET) optimization, to enable the clinical use of proton LATTICE.

**Approach.:**

ARC based proton LATTICE is formulated and solved with energy layer optimization, during which plan quality and delivery efficiency are jointly optimized. In particular, the number of energy jumps (NEJ) is explicitly modelled and minimized during plan optimization for improving delivery efficiency, while target dose conformality and OAR dose objectives are optimized. The plan deliverability is ensured by considering the minimum-monitor-unit (MMU) constraint, and the plan robustness is accounted for using robust optimization. The biological dose is optimized via LET optimization. The optimization solution algorithm utilizes iterative convex relaxation method to handle the dose-volume constraint and the MMU constraint, with spot-weight optimization subproblems solved by proximal descent method.

**Main results.:**

ARC based proton LATTCE substantially improved plan quality from IMPT based proton LATTICE, such as (1) improved conformity index (CI) from 0.47 to 0.81 for the valley target dose and from 0.62 to 0.97 for the peak target dose, (2) reduced esophagus dose from 0.68 Gy to 0.44 Gy (a 12% reduction with respect to 2 Gy valley prescription dose) and (3) improved PVDR from 4.15 to 4.28 in the lung case. Moreover, energy layer optimization improved plan delivery efficiency for ARC based proton LATTICE, such as (1) reduced NEJ from 71 to 56 and (2) reduction of energy layer switching time by 65% and plan delivery time by 52% in the lung case. The biological target and OAR dose distributions were further enhanced via LET optimization. On the other hand, proton ARC LATTCE also substantially improved plan quality from VMAT LATTICE, such as (1) improved CI from 0.45 to 0.81 for the valley target dose and from 0.63 to 0.97 for the peak target dose, (2) reduced esophagus dose from 0.59 Gy to 0.38 Gy (a 10.5% reduction with respect to 2 Gy valley prescription dose) and (3) improved PVDR from 3.88 to 4.28 in the lung case.

**Significance.:**

The feasibility of high-plan-quality proton LATTICE is demonstrated via proton ARC with substantially improved target dose coverage and OAR sparing compared to IMPT, while the plan delivery efficiency for ARC based proton LATTICE can be optimized using energy layer optimization.

## 1. Introduction

The goal of radiation therapy (RT) is to deliver tumoricidal dose while sparing organs-at-risk (OAR). Spatially fractionated RT (SFRT) challenges the conventional RT paradigm by delivering highly modulated and heterogenous spatial dose, that can reduce normal tissue complication and improve tumor control (Prezado 2022). While the SFRT was introduced by Alban Köhler (Laissue et al 2012) over a century ago, GRID (Mohiuddin et al 1990, Zhang et al 2016, Narayanasamy et al 2017) and LATTICE (Wu et al 2010) are two common modern SFRT modalities that have been clinically used to treat patients.

LATTICE can be regarded as a 3D extension of GRID (Wu et al 2010): while GRID has 2D modulated dose distribution of pencils, LATTICE has 3D modulated dose distribution of spheres. LATTICE is more versatile than GRID as it has more treatment planning degrees of freedom for 3D dose shaping in target and sparing of OAR, which is often planned and delivered via photon IMRT and VMAT, while in contrast GRID has 2D spatially-modulated dose with limited dose shaping capability.

While the majority of SFRT is delivered using photons, proton SFRT can be clinically beneficial, since protons have the Bragg peak, which can target and deliver the most dose to tumor target with sharp dose falloff and negligible dose beyond the target. In comparison, photons have a relatively slow decay in depth dose and can create the unwanted dose bath to OAR close to the target. For example, Northwestern Medicine Proton Center developed a proton GRID technique that mimics photon GRID and demonstrated the dosimetric advantages of proton GRID compared to photon GRID, in terms of target dose uniformity and OAR dose reduction (Gao et al 2018, Mohiuddin et al 2020). Henry et al (2017) proposed a proton GRID method utilizing spot scanning and cross-firing techniques, which resulted in improved target coverage and better sparing of OAR compared to the photon GRID. Halthore et al (2023) demonstrated that proton GRID delivers a lower dose to normal tissues and target edges in comparison to VMAT. Grams et al (2022) conducted a dosimetric comparison between the advantage of VMAT LATTICE, brass, and proton GRID techniques. Moreover, the use of protons or particles can further enhance the biological effectiveness of SFRT, which is likely due to immune activation (Potez et al 2019, Tinganelli and Durante 2020).

Proton LATTICE is clinically attractive, because of the potential synergy of the benefit from protons compared to photons, and the benefit from LATTICE compared to GRID. However, there are limited literatures published to date. Mossahebi et al (2024) investigate the robust proton LATTICE demonstrated better dose distribution than the clinically implemented photon lattice technique. Zhang et al (2023c) developed a method for optimizing the positioning of LATTICE vertices, considering both protons and photons, with protons showing superior OAR sparing. This limited work in this area is likely attributed to the fact that proton LATTICE using standard proton RT via intensity modulated proton therapy (IMPT) (with a few beam angles) can be problematic with poor target dose coverage and high dose spill to OAR (Zhang et al 2023c).

In terms of technology readiness, the spot-scanning proton RT is state-of-the-art and highly suitable for delivering SFRT, especially the emerging proton ARC RT (Ding et al 2016). LATTICE is primarily used for bulky tumors where uniform dosing could result in excessive and debilitating radiation toxicity. It has been successfully applied to a wide range of sites, including primary head and neck cancers, intra-abdominal structures, lungs, and sarcomas. The additional degrees of freedom provided by proton ARCs can potentially enhance SFRT plan quality by improving intratumoral sparing, dose conformity, and OAR sparing. Therefore, proton ARCs offer the potential to enhance LATTICE plans for large, deep-seated tumors that are surrounded by critical OARs with dose limitations. This work aims to develop a novel proton LATTICE method using proton ARC (with multiple beam angles) to address the challenges of IMPT in terms of target coverage and OAR sparing, thereby facilitating the clinical application of proton LATTICE.

In addition to plan quality, the plan delivery efficiency will also need to be accounted for during ARC based proton LATTICE, which is unique to proton ARC. The major difference between ARC and IMPT is the need of energy layer optimization for proton ARC. Technically, the energy switch-up time (e.g. 5 s) is much larger than the energy switch-down time (e.g. 0.5 s). While the number of energy jumps (NEJ) may not be an issue for IMPT, because (1) the energy layers are sequenced from high to low for each beam angle, (2) the energy switch-up time (that only occurs during the change of beam angles) overlaps with the beam angle switching time, and (3) IMPT usually has a few beam angles. However, excessive NEJ can substantially increase the plan delivery time for proton ARC, as the gantry is constantly rotating and ARC consists of many beam angles, unless the proper energy layer optimization (Gu et al 2020, Engwall et al 2022, Wuyckens et al 2022, Zhang et al 2022, 2023a) is taken into account for ARC based proton LATTICE.

On the other hand, the biological dose distribution for proton LATTICE needs to be addressed, e.g. to avoid target underdosing or OAR overdosing, for which linear energy transfer (LET) optimization will be considered.

## 2. Methods

### 2.1. Optimization problem formulation

Proton LATTICE treatment planning solves the following optimization problem

(1) $$\begin{array}{l} \underset{x\in {\mathbb{R}}^{N_{x}}}{\min}f(d,\text{Ω})+ELO(x)\\ s.t.\;\left\{\begin{array}{l} x_{j}\in \{0\}\cup [g,+\infty ),j⩽N_{x}\\ d=Ax \end{array},\right. \end{array}$$

where x is the spot weight to be optimized and $N_{x}$ is the number of spots; d is the dose distribution and A is the dose influence matrix with elements $A_{ij}$ referring to the dose received by the ith voxel from the jth spot of the unit weight, i.e. d=Ax; f is the plan-quality optimization objective that will be described shortly in this section; energy layer optimization (ELO) represents the plan-efficiency optimization objective that will be provided in the next section.

In equation (1), the minimum-monitor-unit (MMU) constraint is enforced, i.e. the nonzero spot weight has at least the minimum threshold value g, in order for the plan to be deliverable. Various algorithms have been developed to solve the MMU constraint, including postprocessing methods (Zhu et al 2010, Lin et al 2016, Gao et al 2019) and optimization methods (Albertini et al 2009, Cao et al 2013, Howard et al 2014, Shan et al 2018, Lin et al 2019, Gao et al 2020a). Note that the MMU constraint is nonconvex, especially for large g, for which special optimization methods (Cai et al 2022, Zhu et al 2023) have been developed.

The plan-quality optimization objective f accounts for the differences between the actual dose distribution d and the desirable dose value (e.g. the prescription dose), and clinically-used constraints based on dose-volume-histogram (DVH), i.e.,

(2) $$f(d,\text{Ω})=\sum_{i=1}^{N_{L2}}w_{1,i}{\left\|d_{{\text{Ω}}_{1,i}}-d_{1,i}\right\|}^{2}+\sum_{i=1}^{N_{\text{DVH}-\max}}w_{2,i}{\left\|d_{{\text{Ω}}_{2,i}}-d_{2,i}\right\|}^{2}+\sum_{i=1}^{N_{\text{DVH}-\min}}w_{3,i}{\left\|d_{{\text{Ω}}_{3,i}}-d_{3,i}\right\|}^{2}$$

- The first term denotes the $L_{2}$-type least-square terms for the targets and OAR. It measures the discrepancy between the optimized dose distribution $d_{\text{Ω}}$ and the desirable dose value $d_{1}$. $N_{L2}$ is the number of the $L_{2}$-type objective terms.
- The second term is for DVH-max-dose constraints. The DVH-max-dose constraint is defined by
(3) $$D_{p\text{\%}}⩽c:⩽p\text{\% of ROI receives}\geq c\;\text{dose},$$
  which implies the minimum dose received by the maximally exposed p% of the total ROI volume is at most c. When DVH-max-dose constraint is not fully satisfied, the corresponding active-index set Ω of the violated voxels can be rigorously determined by
(4) $$\text{Ω}=\left\{i|p\;⩽\;i^{\prime }⩽p^{*},d_{p^{*}}^{\prime }=c\right\},\;\text{if}\;d_{p}^{\prime }>c,$$
  where $d^{\prime }$ is the sorted d in descending order. Based on equation (4), the third term of equation (2) penalizes the least-square difference for the dose voxels in Ω between their current dose d and the DVH constraint c, in order to bring these violated dose values under the constraint c, so that the DVH-max-dose constraint can be satisfied.
- The third term is the DVH-min-dose constraint for the targets. The DVH-min-dose constraint is defined by:
(5) $$D_{p\%}⩾c:⩾p\%\;\text{of ROI receives}\;⩾c\;\text{dose,}$$
  which implies the minimum dose received by the maximally exposed p% of the total target volume is at least c. When DVH-min-dose constraint is not fully satisfied, the corresponding active-index set Ω of the violated voxels can be rigorously determined by
(6) $$\text{Ω}=\left\{i|p^{*}⩽i^{\prime }⩽p,d_{p^{*}}^{\prime }=c\right\},\;\text{if}\;d_{p}^{\prime }<c,$$
  where $d^{\prime }$ is the sorted d in descending order. Based on equation (6), the fourth term of equation (2) penalizes the least-square difference for the dose voxels in Ω between their current dose d and the DVH constraint c, in order to bring these violated dose values above the constraint c, so that the DVH-min-dose constraint can be satisfied.

To simplify the notation, the calculation of active-index set Ω via equations (4) and (6) for a given dose distribution d, which in turn depends on x, is denoted by

(7) Ω=H(x).

Note that the dependence of Ω on d or x is nonconvex.

To model the peak and valley target dose for LATTICE, the $L_{2}$ and DVH objective terms in equation (2) are separate for peak target dose region $\left({\text{Ω}}_{\text{peak}}\right)$ and valley target dose region $\left.\left({\text{Ω}}_{\text{valley}}\right.\right)$ respectively, which are treated as two non-overlapping regions for treatment planning purpose. For example, the $L_{2}$-type term in equation (2) becomes

(8) $$w_{\text{peak}}{\left\|d_{{\text{Ω}}_{\text{peak}}}-d_{\text{peak}}\right\|}^{2}+w_{\text{valley}}{\left\|d_{{\text{Ω}}_{\text{valley}}}-d_{\text{valley}}\right\|}^{2},$$

where $d_{\text{peak}}$ and $d_{\text{valley}}$ are the prescription dose for the peak dose and the valley dose respectively.

### 2.2. Energy layer optimization

The plan-efficiency optimization objective ELO(x) in equation (1) for ARC based proton LATTICE is based on the direct minimization of NEJ (Zhang et al 2023a), i.e.

(9) $$\mathrm{ELO}(x)=\lambda \|\mathrm{DVS}(Wx){\|}_{+}^{2},$$

with the following steps.

- W is a linear matrix operator that sums spot weight x of the same energy for each energy to an energy vector $y_{1}=Wx$.
- Since whether the energy is used or not instead of the summed spot weight value is relevant to the NEJ minimization, $y_{1}$ is binarized component-wisely to $y_{2}$, using a sigmoid function transform S, i.e. $y_{2}=2/\left(1+\exp\left(-\alpha y_{1}\right)\right)-1$, where α>0 is a predefined constant that controls the sharpness of sigmoid function transform.
- Next, $y_{2}$ is dot-multiplied with an energy-order vector V to get $y_{3}$, *i.e.* $y_{3,j}=V_{j}y_{2,j}$ for the jth energy, in which the zero elements indicate the unused energy layers.
- Then, the discrete finite-difference operator D is applied to the non-zero elements of $y_{3}$ to identify the energy jumps (i.e. when $Dy_{3}$ is positive), for which ${\left(Dy_{3}\right)}_{j}=y_{3,j-}y_{3,j’}$, where j’ is the index of the next immediate non-zero element adjacent to j. Note that the operation $Dy_{3}$ is nonconvex due to the skip of zero elements.
- Last, the max function ${\left(.\right)}_{+}=\max(.,0)$ selects the energy jumps, which are summed together in equation (9) to arrive at the plan-efficiency optimization objective ELO, with the plan-efficiency regularization parameter λ>0 to balance between f and ELO in equation (1).

### 2.3. Optimization algorithm

The optimization solution algorithm for solving equation (1) will be based on iterative convex relaxation (ICR) method (Gao 2019, Gao et al 2020b, Li et al 2023a, 2023b) to deal with the nonconvexity from DVH constraints (i.e. Ω) in f and the finite-difference operator D in ELO.

For the ease of presentation, equation (1) is reformulated as

(10) $$\begin{array}{l} \underset{x}{\min}\|A(\text{Ω}(x))x-b(\text{Ω}(x)){\|}_{2}^{2}+\lambda \|DVS(Wx){\|}_{+}^{2}.\\ \text{s.t.}\;x\in \{0\}\cup [g,+\infty )\text{.} \end{array}$$

**Algorithm 1.** Proximal gradient descent method for solving equation (13).

**Input** $A\left({\text{Ω}}^{m+1}\right)$, $b\left({\text{Ω}}^{m+1}\right)$, $z_{0}=x^{m}$, MMU threshold g, step size s>0, iteration number K.	
1.	**For** k=1,2,…,K−1
2.	$y_{k+1}=x_{k}-s\left(A{\left({\text{Ω}}^{m+1}\right)}^{T}\left(A\left({\text{Ω}}^{m+1}\right)z_{k}-b\left({\text{Ω}}^{m+1}\right)\right)+2\lambda \max\left(W^{T}S^{\prime }\left(V^{T}DVS\left(Wz_{k}\right),0\right)\right)\right)$,
3.	$z_{k+1}=\left\{\begin{array}{l} \max\left(y_{k+1},g\right),y_{k+1}⩾g/2\\ 0,\text{otherwise} \end{array}\right.$
4.	**End**
**Output:** $x^{m+1}=z_{K}$.	

ICR is used to decouple the nonconvex terms Ω and D with the following alternating updates of terms Ω, D, and x, as the outer loop of the solution algorithm,

(11) $${\text{Ω}}^{m+1}=H\left(x^{m}\right);$$

(12) $$D^{m+1}=I\left(x^{m}\right);$$

(13) $$x^{m+1}=\left\{\begin{array}{l} \underset{x}{\arg\min}{\left\|A\left({\text{Ω}}^{m+1}\right)x-b\left({\text{Ω}}^{m+1}\right)\right\|}_{2}^{2}+\lambda {\left\|D^{m+1}VS(Wx)\right\|}_{+}^{2}\\ \text{s.t.}\;x\in \{0\}\cup \left[g,+\infty \right) \end{array}\right.$$

The update of x in equation (13) is through proximal gradient descent (PGD) method (algorithm 1) (Nesterov 2018), as the inner loop of the solution algorithm. Essentially, PGD alternates between the data-fidelity update using the gradient descent to obtain $y_{k+1}$ (Line 2 of algorithm 1), and the MMU-constraint update using its analytic solution to obtain $z_{k}+1$ (Line 3 of algorithm 1).

### 2.4. LET optimization

This section develops biological dose optimization method for proton ARC based LATTICE using LET optimization, for maximizing biological dose in target or/and minimizing biological dose in OAR. The biological dose is typically linked to the physical dose through the concept of relative biological effectiveness (RBE), which is defined as the ratio of the equivalent photon dose to the physical particle dose. The application of crude constant RBE 1.1 in protons can potentially severely underestimate the LET to the normal tissue of the protons at the end of the Bragg peaks near the target. Therefore, instead of utilizing constant RBE, we will optimize the biological dose for proton ARC based LATTICE via LET optimization, based on our previously established LET optimization framework (Li et al 2023a). For simplicity, let us consider the following LET optimized ARC based LATTICE problem without ELO

(14) $$\begin{array}{l} \underset{x\in {\mathbb{R}}^{N_{x}}}{\min}f(d,\text{Ω})+h(L,\text{Ω})\\ s.t.\;\left\{\begin{array}{l} d=Ax\\ L=\frac{B\circ Ax}{d}\\ x_{j}\in \{0\}\cup [g,+\infty ),j⩽N_{x} \end{array},\right. \end{array}$$

where B is the LET influence matrix, L is the LET dose is computed by the dose averaged LET dose as shown in the second equality of (14), ° denotes the Hadamard product and the division operates element- wisely, and h is the LET optimization objective.

ICR is developed to effectively solve the LET problem equations (15) and (16), during which we decouple the DVH, MMU constraints, and linearize the nonlinear LET term as follows:

(15) $${\text{Ω}}^{m+1}=H\left(x^{m}\right);$$

(16) $$d^{m+1}=Ax^{m};$$

(17) $$x^{m+1}=\left\{\begin{array}{l} \underset{x}{\arg\min}f\left(d,{\text{Ω}}^{m+1}\right)+h\left(\frac{B\circ Ax}{d^{m+1}},{\text{Ω}}^{m+1}\right)\\ \text{s.t.}\;x_{j}\in \{0\}\cup [g,+\infty ),j\leq N_{x} \end{array}\right.$$

The update of active set in (15) is as that in (3)–(7). For (16), with the fixed active set and $d^{m+1}$, the objective becomes convex, which then can be efficiently solved by alternating direction method of multipliers (ADMM) (Boyd et al 2011, Gao 2016).

### 2.5. Materials

We compare three different scenarios of proton LATTICE: (1) IMPT based proton LATTICE (namely ‘IMPT’ in the following), (2) ARC based proton LATTICE (solved by ICR and ADMM (Boyd et al 2011, Gao 2016)) without energy layer optimization (‘ARC’), and (3) ARC based proton LATTICE with ‘ELO’. The total prescribed doses for the abdomen and lung are 20 Gy and 24 Gy, respectively. The centers of the dose peaks were manually selected to achieve the best possible plan quality, with the peak radius set at 7 mm for the abdomen and 9 mm for the lung. For both the abdomen and lung, the peak target dose was set at 10 Gy and valley target dose was set at 2 Gy. OAR bowel large and bowel small were considered in plan objective for abdomen; esophagus, carina and spinal cord were considered for lung. $L_{2}$ type plan objectives were used for all OARs’. D95 ⩾ 100% DVH min plan objectives were used for peak and valley in both cases. The dose influence matrix was generated via MatRad (Wieser et al 2017) with 5 mm lateral spacing on a 3 mm^3^ dose grid. For IMPT, beam angles were (60°, 150°, 240°, 330°) for the abdomen and (0°, 120°, 240°) for the lung. For ARC, the full 360° was used, which was divided into 72 equally segmented 5° angular intervals.

Clinically-used DVH constraints were adopted, and both PTV and CTV treatment planning scenarios were considered for proton LATTICE: (1) with PTV as the planning target, all plans were normalized to $D_{95\%}=100\%$ for valley target dose in PTV; (2) with CTV as the planning target, robust optimization was considered using 5 mm setup uncertainty and 3.5% range uncertainty, via probabilistic formulation and solution algorithm (Unkelbach et al 2018), and all plans were normalized to $D_{95\%}=100\%$ for valley target dose in CTV for the worse-case scenario.

To demonstrate the advantage of ARC in biological dose conformality compared to IMPT, two following scenarios of LET optimization were considered: (1) target LET maximization (‘LET1’); (2) target LET maximization and OAR LET minimization (‘LET2’), where a 1 cm expansion of PTV (‘PTV1cm’) is defined as a high-dose OAR. The LET optimization algorithm is based on ICR (Li et al 2023a). The results are presented in table 4, figures 4 and 5.

To demonstrate the advantage of protons for LATTICE compared to photons, two following scenarios of photons were considered: (1) photon planning with the same number of beam angles as IMPT (‘IMRT’); (2) proton planning with the same number of beam angles as ARC (‘VMAT’). The results are presented in table 5 and figure 6.

To quantify the plan quality, (1) the conformity index (CI) was defined as $V2\;100/\left(V\times V^{\prime }\;100\right)\left(V_{100}\right.$: target volume receiving at least 100% of prescription dose; V: target volume; $V^{\prime }\;100$: total volume receiving at least 100% of prescription dose), for the valley dose $\left({\text{CI}}_{v}\right)$ and the peak dose $\left({\text{CI}}_{p}\right)$ respectively; (2) peak-to-valley dose ratio (PVDR) was calculated as the ratio of mean peak dose over mean valley dose.

To quantify the delivery efficiency for ARC plans, the energy switching time $T_{E}$ and the spot delivery time $T_{B}$ were calculated: (1) $T_{E}=\text{NEJ}\cdot t_{1}+\text{NED}\cdot t_{2}$, where NEJ and $t_{1}=5.5\;s$ are the number and time of energy switch-up, and NED and $t_{1}=0.6\;s$ are the number and time of energy switch-down; (2) $T_{B}=\|x{\|}_{1}/\gamma$, where $\|x{\|}_{1}$ is the sum of beam weights and $\gamma =2.6\times 10^{10}$ protons per minute that corresponds to the MMU threshold $g=5\times 10^{6}$ protons used in this work.

## 3. Results

IMPT, ARC, and ELO are compared in terms of both plan quality and plan delivery efficiency, for the scenarios with PTV planning, CTV planning, and multi-criteria optimization (MCO) respectively in the following sections.

### 3.1. PTV planning

ARC improved plan quality from IMPT. As shown in table 1, compared to IMPT, (1) ARC decreased the plan-quality objective from 344.6–131.1, improved the target dose conformality, i.e. ${\text{CI}}_{v}$ from 0.55 to 0.88, and ${\text{CI}}_{p}$ from 0.44 to 0.68, and reduced OAR dose, e.g. the mean stomach dose from 0.70 Gy to 0.47 Gy, for the abdomen case; (2) ARC decreased the plan-quality objective from 139.0–49.7, improved the target dose conformality, i.e. ${\text{CI}}_{v}$ from 0.47 to 0.80, and ${\text{CI}}_{p}$ from 0.62 to 0.97, and reduced OAR dose, e.g. the mean esophagus dose from 0.68 Gy to 0.44 Gy, for the lung case.

With comparable plan quality, ELO improved plan delivery efficiency from ARC. As shown in table 1, compared to ARC, (1) ELO had decreased NEJ from 71 to 50, and a 56% reduction of energy switching time from 807.5 s to 354.9 s, for the abdomen case; (2) ELO had decreased NEJ from 71 to 56, and a 65% reduction of energy switching time from 1169.3 s to 408.5 s, for the lung case.

**Figure 1.**
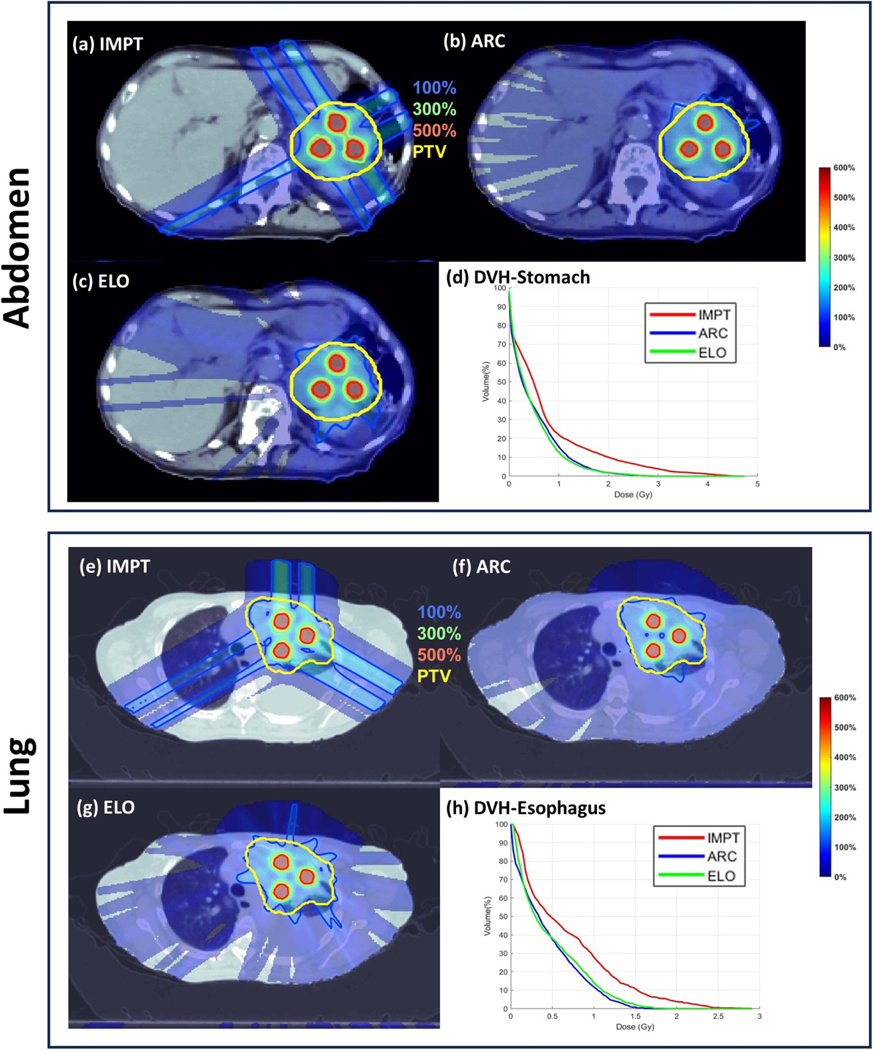
PTV planning. (a)–(d) IMPT dose, ARC dose, ELO dose, and DVH plots for abdomen; (e)–(h) IMPT dose, ARC dose, ELO dose, and DVH plots for lung. The dose plot window is [0%, 600%] of the valley prescription dose. 100%, 300%, 500% isodose lines and PTV are highlighted in dose plots.

The plots of dose distributions and OAR DVH are provided in figure 1, which also shows that (1) ARC improved plan quality from IMPT, and (2) ELO had similar plan quality with ARC.

### 3.2. CTV planning with robust optimization

**Figure 2.**
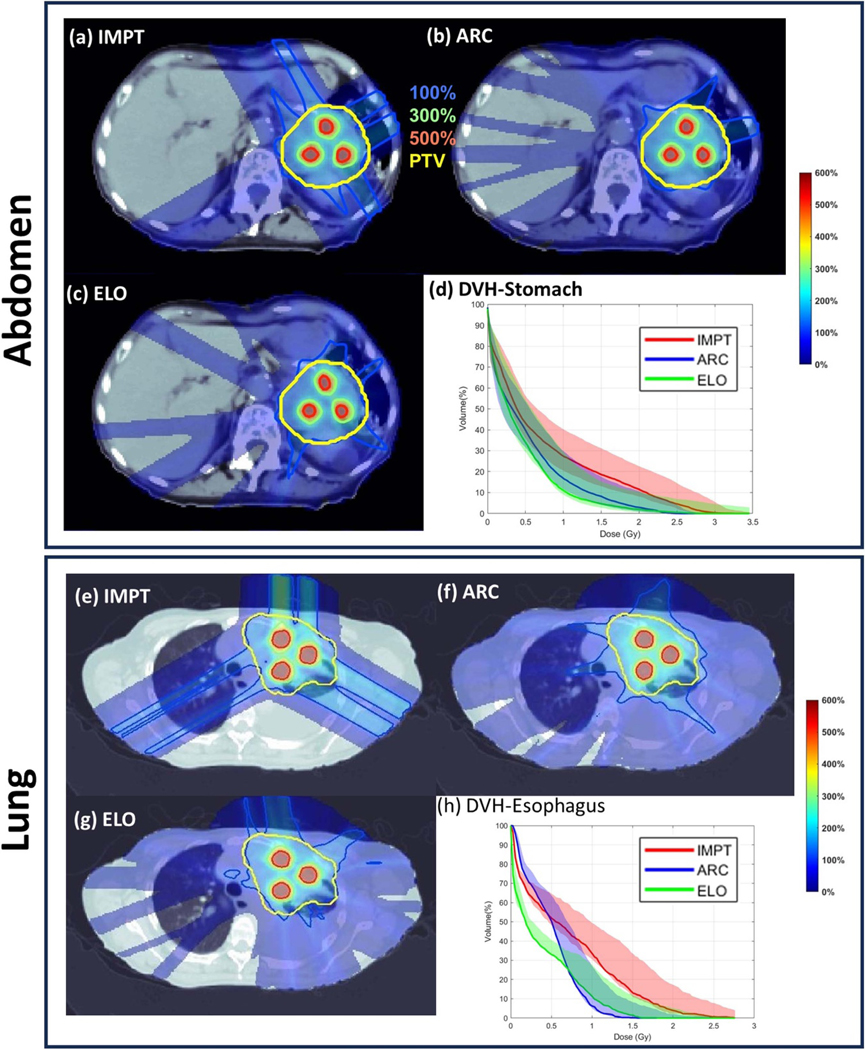
CTV planning with robust optimization. (a)–(d) IMPT dose, ARC dose, ELO dose, and DVH plots for abdomen; (e)–(h) IMPT dose, ARC dose, ELO dose, and DVH plots for lung. The dose plot window is [0%, 600%] of the valley prescription dose. 100%, 300%, 500% isodose lines and PTV are highlighted in dose plots. The solid DVH curves are from the nominal scenario.

The dosimetric parameters (CI, PVDR and ${\text{D}}_{\text{OAR}}$) presented in table 2 are calculated based on the total probabilistically-weighted dose distribution from all uncertainty scenarios. The dose plots in figure 2 are from the total probabilistically-weighted dose distribution from all uncertainty scenarios, and the DVH plots in figure 2 include all uncertainty scenarios, with solid lines representing the nominal scenario.

Compared to IMPT, ARC improved plan quality, as shown in table 2. For the abdomen case, ARC reduced the plan-quality objective from 167.0 (worst-case 367.8, best-case 146.8) to 118.3 (worst-case 267.5, best-case 87.2). It also improved the target dose conformality, with ${\text{CI}}_{v}$ increasing from 0.61 (worst-case 0.41) to 0.77 (worst-case 0.59, best-case 0.78), and ${\text{CI}}_{p}$ increasing from 0.43 (worst-case 0.24) to 0.51 (worst-case 0.28, best-case 0.64). Furthermore, the mean stomach dose decreased from 0.69 Gy (worst-case 1.07 Gy, best-case 0.59 Gy) to 0.51 Gy (worst-case 0.78 Gy, best-case 0.40 Gy). Similarly, for the lung case, ARC decreased the plan-quality objective from 186.8 (worst-case 861.8) to 115.6 (worst-case 361.3, best-case 108.6). The ${\text{CI}}_{v}$ improved from 0.44 (worst-case 0.34) to 0.68 (worst-case 0.53, best-case 0.70), and ${\text{CI}}_{p}$ increased from 0.39 (worst-case 0.19) to 0.40 (worst-case 0.25, best-case 0.44). Additionally, the mean esophagus dose was reduced from 0.71 Gy (worst-case 1.04 Gy, best-case 0.69 Gy) to 0.54 Gy (worst-case 0.73 Gy, best-case 0.49 Gy). These results demonstrate ARC’s advantage in improving dose distribution and reducing OAR exposure compared to IMPT.

With comparable plan quality, ELO improved plan delivery efficiency from ARC. As shown in table 2, compared to ARC, (1) ELO had decreased NEJ from 71 to 53, and a 57% reduction of energy switching time from 874.0 s to 379.9 s, for the abdomen case; (2) ELO had decreased NEJ from 71 to 49, and a 71% reduction of energy switching time from 1154.3 s to 328.9 s, for the lung case.

The plots of dose distributions and OAR DVH provided in figure 2 and histogram plots of quantitative value of different methods given in figure S1 also show that (1) ARC improved plan quality from IMPT, and (2) ELO had similar plan quality with ARC.

### 3.3. Multi-criteria optimization

**Figure 3.**
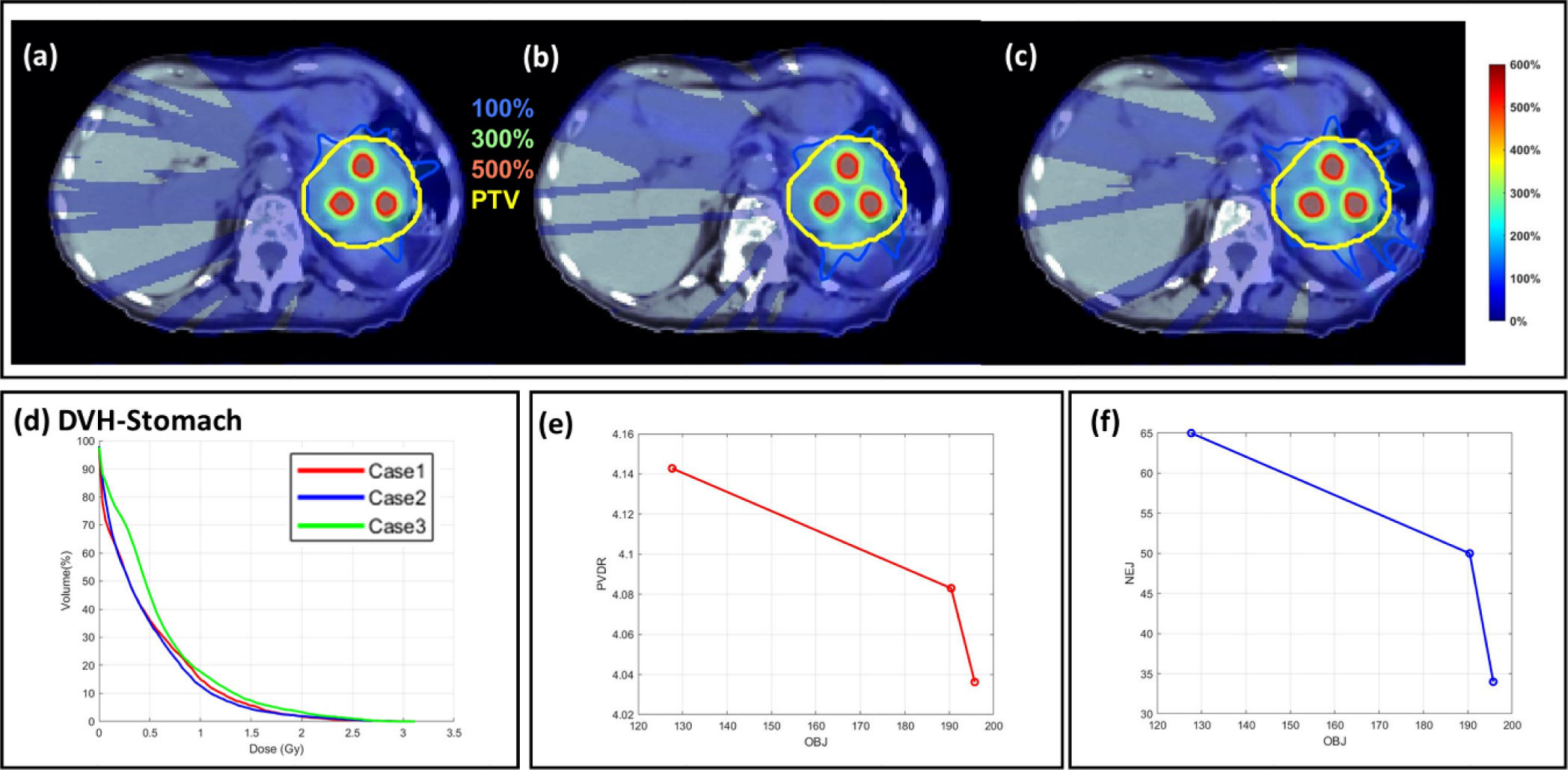
Quality-efficiency multi-criteria optimization. Dose (a)–(c) and DVH (d) plots for Case 1–3 that correspond to 3 MCO plans in table 3. The dose plot window is [0%, 600%] of the valley prescription dose. 100%, 300%, 500% isodose lines and PTV are highlighted in dose plots. (e) PVDR *v.s.* f; (f) NEJ *v.s.* f.

MCO plans are compared to evaluate the trade-off between plan quality and delivery efficiency, by varying the weight λ of energy layer optimization in equation (9). The abdomen MCO results are provided in table 3 and figure 3. The value of λ increased from Case 1 to Case 3, with reduced plan quality, and yet improved delivery efficiency. Case 2 was identical to ELO in table 1 and figure 1.

### 3.4. LET optimization

**Figure 4.**
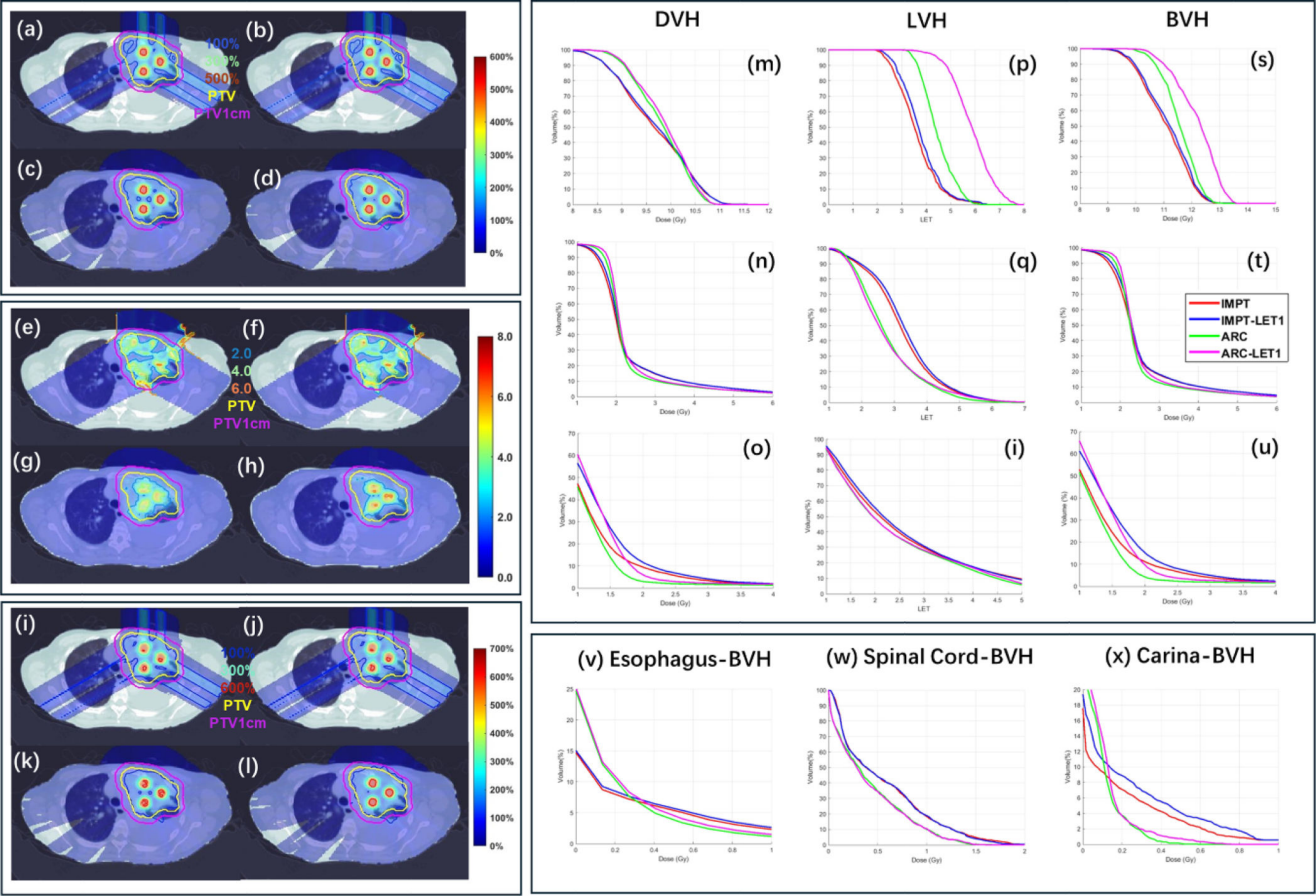
LET optimization (target only). Physical dose (a)–(d), LET (e)–(h) and biological dose (i)–(l) of IMPT, IMPT-LET1, ARC, and ARC-LET1. Physical DVH (m)–(o), LET-volume histogram (LVH) (p)–(r), and biological DVH (BVH) (s)–(u) of peak, valley and PTV1cm. BVH plots of esophagus, spinal cord and carina are presented in (v)–(x). For physical dose (a)–(d), the dose plot window is [0%, 600%] of the valley prescription dose, and 100%, 300%, 500% isodose lines, PTV and PTV1cm are highlighted in dose plots. For LVH, the plot window is [0.0,8.0], and 2.0,4.0, 6.0 isolines, PTV and PTV1cm are highlighted in the plots. For biological dose (e)–(h), the dose plot window is [0%, 700%] of the valley prescription dose, and 100%, 300%, 600% isodose lines, PTV and PTV1cm are highlighted in dose plots.

**Figure 5.**
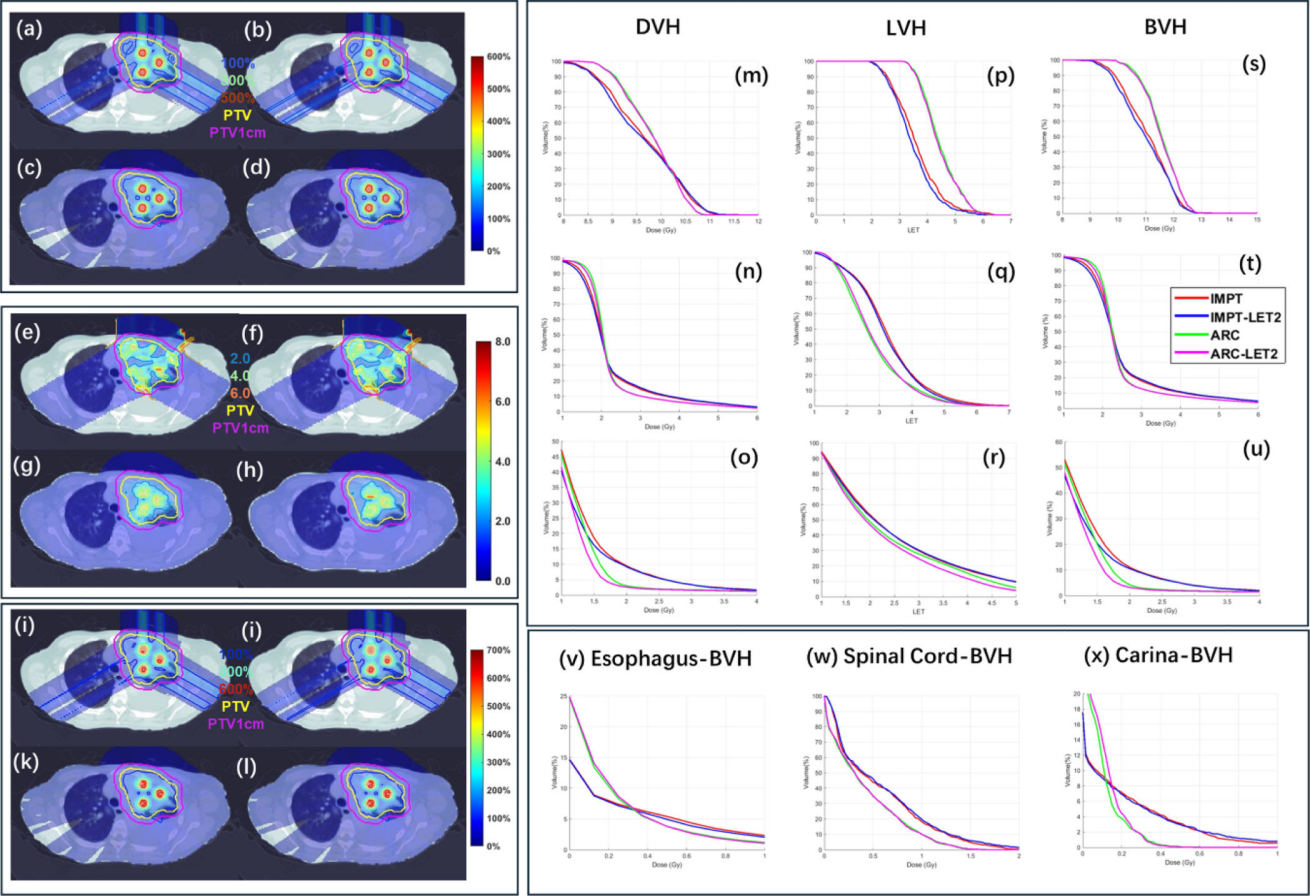
LET optimization (target and PTV1cm). Physical dose (a)–(d), LET (e)–(h) and biological dose (i)–(l) of IMPT, IMPT-LET2, ARC, and ARC-LET2. Physical DVH (m)–(o), LET-volume LVH (p)–(r), and BVH (s)–(u) of peak, valley and PTV1cm. BVH plots of esophagus, spinal cord and carina are presented in (v)–(x). For physical dose (a)–(d), the dose plot window is [0%, 600%] of the valley prescription dose, and 100%, 300%, 500% isodose lines, PTV and PTV1cm are highlighted in dose plots. For LVH, the plot window is [0.0,8.0], and 2.0,4.0, 6.0 isolines, PTV and PTV1cm are highlighted in the plots. For biological dose (e)–(h), the dose plot window is [0%, 700%] of the valley prescription dose, and 100%, 300%, 600% isodose lines, PTV and PTV1cm are highlighted in dose plots.

The results are presented in table 4 and figures 4 and 5. It was observed that, without LET optimization, ARC achieved better target coverage than IMPT (table 4 and figures 4 (a), (c), (e), (g), (i) and (k)), as well as DVH, LET-volume histogram (LVH), and biological DVH (BVH) plots from (m) to (x)). With target LET maximization (i.e. LET1), ARC-LET1 showed clear improvements in LET (figure 4 (p)) and biological dose (figure 4 (s)) compared to ARC. The valley LET and biological dose, however, remained nearly the same between ARC-LET1 and ARC (figures 4 (q) and (t)). The LET maximization in the target resulted in slightly degraded LET and biological dose in the PTV1cm, as shown in figures 4 (i) and (u). Therefore, we further explored a joint optimization approach of target LET maximization and PTV1cm LET minimization (i.e. LET2). Figure 5 demonstrates that ARC-LET2 achieves comparable peak and valley LET, as well as biological dose, compared to ARC (figures 5(p), (q), (s) and (t)), while achieving lower LET values and biological dose in PTV1cm (figures 5(i) and (u)). Importantly, LET optimization did not significantly compromise OAR sparing in both IMPT and ARC. For instance, the mean biological doses to the esophagus, spinal cord, and carina with LET optimization were nearly identical to those without LET optimization (table 4). However, ARC exhibited relatively better results, e.g. ARC-LET2 decreases the mean biological doses to the esophagus, spinal cord, and carina decreased from 0.57, 0.04, and 0.18–0.42, 0.03, and 0.10, respectively.

### 3.5. Photons v.s. protons

**Figure 6.**
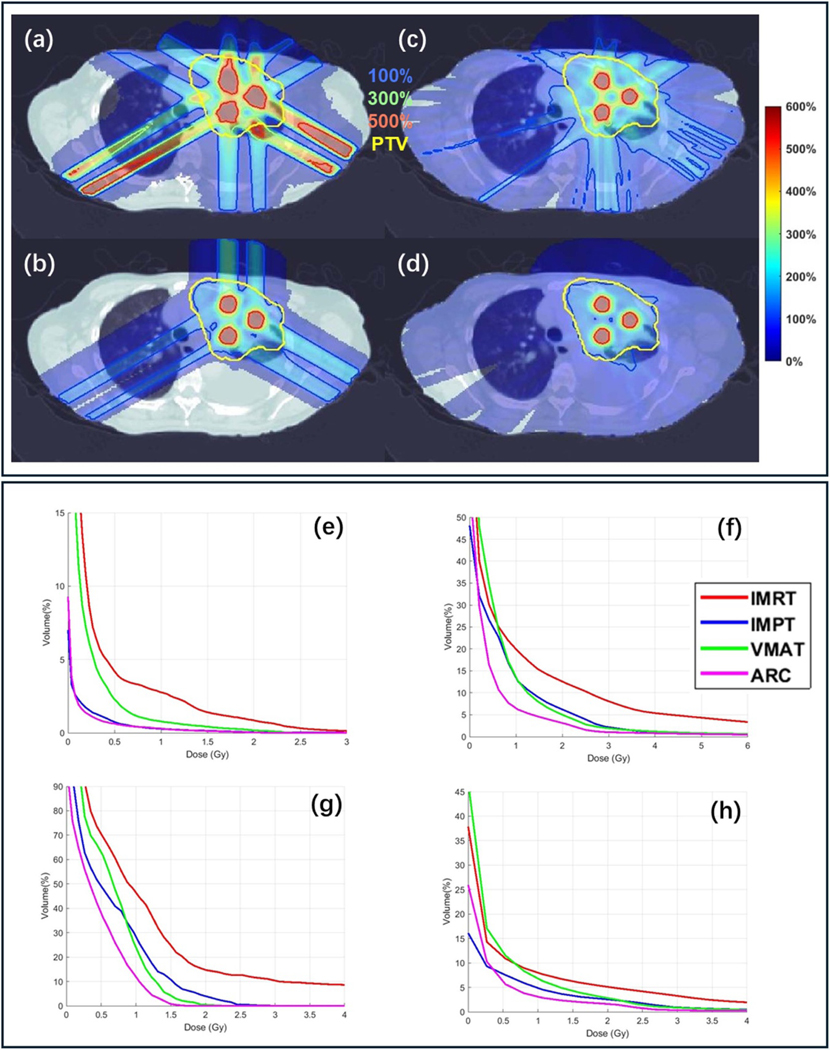
Protons v.s. photons. Dose plots (a)–(d) of IMRT, IMPT, VMAT, and ARC. (e)–(h): DVH of heart, lung, esophagus and body. The dose plot window for physical dose is [0%, 600%] of the valley prescription dose. 100%, 300%, 500% isodose lines and PTV are highlighted in dose plots.

This section will demonstrate the benefit of protons over photons for LATTICE. The results are shown in table 5 and figure 6. First, we compared IMRT (figure 6(a)) and IMPT (figure 6(b)) with the same number of beam angles, and then VMAT (figure 6(c)) and ARC (figure 6(d)) again with the same number of beam angles. Both of these two comparisons indicate the benefit of protons over photons, such as IMPT had lower objective value, higher conformal index for peak and valley regions, higher PVDR and lower OAR mean dose than IMRT. Compared with VMAT based LATTICE and IMPT, proton ARC based LATTICE had substantially better target coverage and OAR sparing. For example, the objective value for ARC was 49.7 v.s. 138.9 and 62.9 in VMAT and IMPT; the CI for peak and valley region were 0.81 and 0.97 in ARC while they were 0.48, 0.62 in IMPT and 0.45, 0.63 in VMAT. ARC obtained higher PVDR (4.28 v.s. 4.16 and 3.88) and lower mean dose to the OAR (0.25 v.s. 0.33 and 0.42).

## 4. Discussion

In this study, we developed a proton ARC method for LATTICE. Compared to IMPT, ARC can leverage the benefits of the increased degree of freedom to improve target coverage and OAR sparing. To improve the delivery of efficiency of ARC, ELO is developed for LATTICE that jointly optimize plan quality and delivery efficiency. Comparative LATTICE treatment planning studies show that ARC can achieve more conformal dose distribution, better OAR sparing, and higher PVDR, compared to IMPT, IMRT and VMAT, with a thorough comparison of dosimetric values recommended by the RSS SFRT working group (Zhang et al 2020) in supplementary material).

Compared to ARC, the utility of ELO can reduce the delivery time at the expense of slightly degraded plan quality. For proton ARC LATTICE, it is practically appealing to ensure the plan quality while having the fast delivery, for which ELO utilizes novel energy layer regularizer to characterize the energy jumps and incorporates it into the plan objective to build an ARC LATTICE optimization model that accounts both plan quality and delivery efficiency. The trade-off between plan quality and delivery efficiency for ELO is evaluated by varying the regularization parameter λ in equation (2). Although the optimal value of λ should be case-dependent and may require manual tuning, a sufficiently good value of λ in terms of plan quality that is generally applicable has been identified for our codes. On the other hand, the optimization algorithm for the hyperparameter λ can be explored, via Bayesian optimization and our previous method (Gao 2016).

The delivery time for ARC based LATTICE (without ELO) was approximately 15–20 min (tables 1–3). With ELO, the delivery time was halved, which was comparable with IMPT. However, ELO exhibits a significantly improved dose distribution compared to IMPT. Here the delivery time only consists of two major components: spot delivery time and energy switching time. For more accurate estimation, spots traveling time, and gantry speed should be considered as well (Liu et al 2021).

Regarding the integral dose comparison between IMPT and ARC, ARC exhibits higher dose in low-dose volumes, but lower dose in high-dose volumes, compared to IMPT, e.g. as shown in figure 6(h), while ARC has lower total integral dose than IMPT, e.g. a reduction from 0.11 Gy to 0.1 Gy as shown in table 5.

Regarding the robustness to intranational motion, the proper setup uncertainties and robust optimization method have been accounted for. Moreover, the implementation of immobilization, in-room image guidance, and spirometer motion management strategies should minimize setup and motion uncertainties. On the other hand, the motion-robust ARC LATTICE approach could be developed in the future work, for which each peak vertex is covered by one and the only one field in order to minimize the dose delivery time to each peak vertex.

Note that since IMPT is delivered with only a few beams, the choice of beam angles can greatly alter the plan quality. The beam angles can be optimized through beam angle optimization (BAO) (Cao et al 2012, Gu et al 2018, Shen et al 2023). Although BAO is not used in this work, the choice of beam angles in this work is based on clinically used standard template at our institution and therefore should be a reasonably good choice.

Although the exact mechanism behind SFRT is still unknown, the heterogenous dose distribution may be attributed to a sequence of interrelated events (Potez et al 2019, Tinganelli and Durante2020): (1) The tumor’s blood supply undergoes a diminution due to vascular disruption and subsequent occlusion; (2) the tumor endothelial cells, compromised by radiation, act as gateways for the circulating inflammatory and immune cells; (3) SFRT brings a decrease in proliferation index which correlates with the induction of tumor senescence; (4) the senescent cells appear to excrete cytokines, precipitating the infiltration of various immune cell types. These cells play a pivotal role in the elimination of senescent cells, thereby contributing significantly to the suppression of tumor growth.

PVDR is a key parameter that distinguishes SFRT from conventional radiotherapy. In LATTICE, PVDR is calculated as the ratio of mean peak target dose over mean valley target dose. In this work, PVDR is not explicitly optimized during treatment planning; instead, the dose objectives for peak and valley dose are enforced to minimize the difference between actual peak (valley) dose and the prescribed peak (valley) dose respectively, and then PVDR is calculated after plan optimization. However, it is demonstrated that PVDR optimization can be incorporated into plan optimization to jointly optimize PVDR and dose objectives (Zhang et al 2023b), which can be used to further improve PVDR while preserving the plan quality.

On the other hand, the lattice positioning is a key parameter that can impact the optimality of PVDR and dose objective. Although currently lattice positions are manually determined before treatment planning, the lattice positioning should be optimized during treatment planning, since the optimal lattice positions cannot be determined before treatment planning and have to be iteratively solved during treatment planning based on joint optimization of PVDR and dose objectives (Zhang et al 2023c). A future work is to investigate the joint optimization of lattice positions and spot weights for proton LATTICE.

Despite of potential biological enhancement of protons (Potez et al 2019, Tinganelli and Durante 2020) and improved plan quality of protons (section 3.5) compared to photons, the clinical advantage of protons for LATTICE to photons remains to be seen. Note proton SFRT is still in its early stage, with very limited proton GRID clinical studies (Mohiuddin et al 2020), and no proton LATTICE clinical study to date. Therefore, this work is timely as it provides proton LATTICE plan optimization and treatment planning methods that are essential for the clinical use of proton LATTICE. Furthermore, the clinical use of SFRT is expanding from the treatment of bulky advanced tumors to a broad variety of disease sites, with increasingly curative-intent treatment of nonmetastatic primary malignancies, according to a 2024 clinical practice survey conducted by the RSS SFRT working group (Mayr et al 2024), for which proton LATTICE is expected to play a role.

In addition to using protons, the application of heavier ions such as helium and carbon for ARC LATTICE holds significant potential. Helium offers superior dose distribution than protons due to their reduced scattering, which minimizes the impact on surrounding healthy tissues when considering spatially modulated high dose in vertices. The carbon ions, on the other hand, have sharper Bragg peak than protons, which allows for extremely precise dose shaping within the tumor and reduces the risk of radiation exposure to nearby critical structures. In terms of biological effectiveness, both helium and carbon provide higher RBE. This higher RBE can give better tumor control and is especially beneficial for treating radioresistant tumors.

## 5. Conclusion

The feasibility of high-plan-quality proton LATTICE is demonstrated via proton ARC with substantially improved target dose coverage and OAR sparing compared to IMPT. The plan delivery efficiency for ARC based proton LATTICE can be optimized using energy layer optimization.

## Supplementary Material

Supplementary Data

Supplementary material for this article is available online

## Figures and Tables

**Table 1.** PTV planning. The plan-quality dosimetric quantities from left to right: optimization objective value f, CI for valley dose ${\text{CI}}_{v}$, CI for peak dose ${\text{CI}}_{p}$, PVDR, and mean OAR dose ${\text{D}}_{\text{OAR}}$. OAR refers to stomach in the abdomen case and esophagus in the lung case. The plan-efficiency parameters from left to right: NEJ, total number of energy layers (with nonzero spots) $N_{E}$, total number of angles (with nonzero spots) $N_{A}$, energy switching time $T_{E}$, and spot delivery time $T_{B}$. The unit of dose is Gy, and the unit of time is second.

Abdomen	Quality		f	${\text{CI}}_{v}$	${\text{CI}}_{p}$	PVDR	${\text{D}}_{\text{OAR}}$

		IMPT	344.6	0.55	0.44	4.12	0.70
		ARC	131.1	0.88	0.68	4.20	0.47
		ELO	190.4	0.83	0.56	4.08	0.46

	Efficiency		NEJ	$N_{E}$	$N_{A}$	$T_{E}$	$T_{B}$

		ARC	71	767	72	807.5	155.8
		ELO	50	176	61	354.9	166.0
Lung	Quality		f	${\text{CI}}_{v}$	${\text{CI}}_{p}$	PVDR	${\text{D}}_{\text{OAR}}$

		IMPT	139.0	0.47	0.62	4.15	0.68
		ARC	49.7	0.81	0.97	4.28	0.44
		ELO	97.3	0.74	0.77	4.22	0.47

	Efficiency		NEJ	$N_{E}$	$N_{A}$	$T_{E}$	$T_{B}$

		ARC	71	1370	72	1169.3	329.9
		ELO	56	200	70	408.5	362.4

**Table 2.** CTV planning with robust optimization. The plan-quality dosimetric quantities from left to right: optimization objective value f, CI for valley dose ${\text{CI}}_{v}$, CI for peak dose ${\text{CI}}_{p}$, PVDR, and mean OAR dose ${\text{D}}_{\text{OAR}}$. OAR refers to stomach in the abdomen case and esophagus in the lung case. The plan-efficiency parameters from left to right: number of energy jumps NEJ, total number of energy layers (with nonzero spots) $N_{E}$, total number of angles (with nonzero spots) $N_{A}$, energy switching time $T_{E}$, and spot delivery time $T_{B}$. The unit of dose is Gy, and the unit of time is second.

Abdomen	Quality		f	${\text{CI}}_{v}$	${\text{CI}}_{p}$	PVDR	${\text{D}}_{\text{OAR}}$

		IMPT	167.0	0.61	0.43	3.60	0.69
		ARC	118.3	0.77	0.51	3.62	0.51
		ELO	124.3	0.76	0.53	3.65	0.59

	Efficiency		NEJ	$N_{E}$	$N_{A}$	$T_{E}$	$T_{B}$
		ARC	71	878	72	874.0	184.0
		ELO	53	185	64	379.9	185.0
Lung	Quality		f	${\text{CI}}_{v}$	${\text{CI}}_{p}$	PVDR	${\text{D}}_{\text{OAR}}$

		IMPT	186.8	0.44	0.39	3.72	0.71
		ARC	115.6	0.68	0.40	3.38	0.54
		ELO	126.0	0.72	0.47	3.75	0.41

	Efficiency		NEJ	$N_{E}$	$N_{A}$	$T_{E}$	$T_{B}$

	ARC	71	1345	72	1154.3	443.3
	ELO	49	149	60	328.9	411.2

**Table 3.** Quality-efficiency multi-criteria optimization. Case 1–3 are 3 MCO abdomen plans with tradeoffs in plan efficiency and plan quality. The plan-quality dosimetric quantities from left to right: optimization objective value f, CI for valley dose ${\text{CI}}_{v}$, CI for peak dose ${\text{CI}}_{p}$, PVDR, and mean stomach dose ${\text{D}}_{\text{OAR}}$. The plan-efficiency parameters from left to right: number of energy jumps NEJ, total number of energy layers (with nonzero spots) $N_{E}$, total number of angles (with nonzero spots) $N_{A}$, energy switching time $T_{E}$, and spot delivery time $T_{B}$. The unit of dose is Gy, and the unit of time is second.

	f	${\text{CI}}_{v}$	${\text{CI}}_{p}$	PVDR	${\text{D}}_{\text{OAR}}$

Case 1	127.7	0.86	0.67	4.14	0.48
Case 2	190.4	0.83	0.56	4.08	0.46
Case 3	195.7	0.78	0.52	4.04	0.59

	NEJ	$N_{E}$	$N_{A}$	$T_{E}$	$T_{B}$

Case 1	65	557	70	657.0	156.7
Case 2	50	176	61	354.9	166.0
Case 3	34	93	52	246.3	186.5

**Table 4.** LET optimization. The plan-quality dosimetric quantities from left to right: optimization objective value f, CI for valley dose ${\text{CI}}_{v}$, CI for peak dose ${\text{CI}}_{p}$, PVDR, mean biological esophagus dose ${\text{D}}_{\text{Eso}}$, mean biological spinal cord dose ${\text{D}}_{\text{SC}}$, mean biological carina dose ${\text{D}}_{\text{Ca}}$, mean biological target dose ${\text{B}}_{\text{PTV1cm}}$, mean PTV1cm dose ${\text{D}}_{\text{PTV1cm}}$, and mean biological PTV1cm dose ${\text{B}}_{\text{PTV1cm}}$. LET1: target LET maximization; LET2: target LET maximization and PTV1cm LET minimization.

	f	${\text{CI}}_{v}$	${\text{CI}}_{p}$	PVDR	${\text{B}}_{\text{Eso}}$	${\text{B}}_{\text{SC}}$	${\text{B}}_{\text{Ca}}$	${\text{B}}_{\text{PTV}}$	${\text{D}}_{\text{PTV1cm}}$	${\text{B}}_{\text{PTV1cm}}$
IMPT	24.1	0.26	0.37	4.15	0.55	0.04	0.17	2.92	1.10	1.21
IMPT-LET1	23.9	0.28	0.37	4.10	0.55	0.05	0.17	2.97	1.24	1.36
IMPT-LET2	28.7	0.25	0.37	4.15	0.57	0.04	0.18	2.90	1.03	1.13
ARC	15.9	0.52	0.42	4.28	0.41	0.03	0.10	2.87	1.03	1.12
ARC-LET1	19.8	0.56	0.45	4.19	0.41	0.03	0.10	2.97	1.23	1.35
ARC-LET2	18.4	0.48	0.42	4.34	0.42	0.03	0.10	2.84	0.97	1.06

**Table 5.** Protons v.s. photons. The plan-quality dosimetric quantities from left to right: optimization objective value f, CI for valley dose ${\text{CI}}_{v}$, CI for peak dose ${\text{CI}}_{p}$, PVDR, mean heart dose ${\text{D}}_{\text{heart}}$, mean lung dose ${\text{D}}_{\text{lung,}}$, mean esophagus dose ${\text{D}}_{\text{eso,}}$, and mean body dose ${\text{D}}_{\text{body,}}$.

	f	${\text{CI}}_{\text{v}}$	${\text{CI}}_{\text{p}}$	PVDR	${\text{D}}_{\text{heart}}$	${\text{D}}_{\text{lung}}$	${\text{D}}_{\text{eso}}$	${\text{D}}_{\text{body}}$
IMRT	428.1	0.24	0.09	3.74	0.06	0.53	0.90	0.20
IMPT	138.9	0.48	0.62	4.16	0.01	0.33	0.51	0.11
VMAT	62.9	0.45	0.63	3.88	0.05	0.42	0.59	0.18
ARC	49.7	0.81	0.97	4.28	0.01	0.25	0.38	0.10

## Data Availability

The data cannot be made publicly available upon publication because they contain sensitive personal information. The data that support the findings of this study are available upon reasonable request from the authors.

## References

[R1] Albertini F, Gaignat S, Bosshardt M and Lomax AJ 2009 Planning and optimizing treatment plans for actively scanned proton therapy Biomedical Mathematics Promising Directions in Imaging, Therapy Planning and Inverse Problems (Medical Physics Pub Corp) pp 1–18

[R2] Boyd S, Parikh N, Chu E, Peleato B and Eckstein J 2011 Distributed optimization and statistical learning via the alternating direction method of multipliers Found. Trends Mach. Learn 3 1–122

[R3] Cai JF, Chen RC, Fan J and Gao H 2022 Minimum-monitor-unit optimization via a stochastic coordinate descent method Phys. Med. Biol 67 015009 10.1088/1361-6560/ac4212 PMC9295687 34891150

[R4] Cao W, Lim GJ, Lee A, Li Y, Liu W, Ronald Zhu X and Zhang X 2012 Uncertainty incorporated beam angle optimization for IMPT treatment planning Med. Phys 39 5248–56 22894449 10.1118/1.4737870 PMC3422361

[R5] Cao W, Lim G, Li X, Li Y, Zhu XR and Zhang X 2013 Incorporating deliverable monitor unit constraints into spot intensity optimization in intensity-modulated proton therapy treatment planning Phys. Med. Biol 58 5113–25 23835656 10.1088/0031-9155/58/15/5113 PMC3947922

[R6] Ding X, Li X, Zhang JM, Kabolizadeh P, Stevens C and Yan D 2016 Spot-scanning proton arc (SPArc) therapy: the first robust and delivery-efficient spot-scanning proton arc therapy Int. J. Radiat. Oncol. Biol. Phys 96 1107–16 27869083 10.1016/j.ijrobp.2016.08.049

[R7] Engwall E, Battinelli C, Wase V, Marthin O, Glimelius L, Bokrantz R, Fredriksson A and Fredriksson A 2022 Fast robust optimization of proton PBS arc therapy plans using early energy layer selection and spot assignment Phys. Med. Biol 67 065010 10.1088/1361-6560/ac55a6 35172282

[R8] Gao H 2016 Robust fluence map optimization via alternating direction method of multipliers with empirical parameter optimization Phys. Med. Biol 61 2838 26987680 10.1088/0031-9155/61/7/2838

[R9] Gao H 2019 Hybrid proton-photon inverse optimization with uniformity-regularized proton and photon target dose Phys. Med. Biol 64 105003 30978714 10.1088/1361-6560/ab18c7

[R10] Gao H, Clasie B, Liu T and Lin Y 2019 Minimum MU optimization (MMO): an inverse optimization approach for the PBS minimum MU constraint Phys. Med. Biol 64 125022 31082813 10.1088/1361-6560/ab2133

[R11] Gao H, Clasie B, McDonald M, Langen KM, Liu T and Lin Y 2020a Plan-delivery-time constrained inverse optimization method with minimum-MU-per-energy-layer (MMPEL) for efficient pencil beam scanning proton therapy Med. Phys 47 3892–7 32614472 10.1002/mp.14363

[R12] Gao H, Lin B, Lin Y, Fu S, Langen K, Liu T and Bradley J 2020b Simultaneous dose and dose rate optimization (SDDRO) for FLASH proton therapy Med. Phys 47 6388–95 33068294 10.1002/mp.14531

[R13] Gao M, Mohiuddin MM, Hartsell WF and Pankuch M 2018 Spatially fractionated (GRID) radiation therapy using proton pencil beam scanning (PBS): feasibility study and clinical implementation Med. Phys 45 1645–53 29431867 10.1002/mp.12807

[R14] Grams MP, Tseung HSWC, Ito S, Zhang Y, Owen D, Park SS and Ma DJ 2022 A Dosimetric comparison of lattice, brass, and proton grid therapy treatment plans Pract. Radiat. Oncol 12 e442–52 35417782 10.1016/j.prro.2022.03.005

[R15] Gu W, O’Connor D, Nguyen D, Yu VY, Ruan D, Dong L and Sheng K 2018 Integrated beam orientation and scanning-spot optimization in intensity-modulated proton therapy for brain and unilateral head and neck tumors Med. Phys 45 1338–50 29394454 10.1002/mp.12788 PMC5904040

[R16] Gu W, Ruan D, Lyu Q, Zou W, Dong L and Sheng K 2020 A novel energy layer optimization framework for spot-scanning proton arc therapy Med. Phys 47 2072–84 32040214 10.1002/mp.14083 PMC7234928

[R17] Halthore A, Fellows Z, Tran A, Deville C Jr, Wright JL, Meyer J and Sheikh K 2023 Treatment planning of bulky tumors using pencil beam scanning proton GRID therapy Int. J. Part. Ther 9 40–49 36721485 10.14338/IJPT-22-00028 PMC9875826

[R18] Henry T, Ureba A, Valdman A and Siegbahn A 2017 Proton grid therapy: a proof-of-concept study Technol. Cancer Res. Treat 16 749–57 28592213 10.1177/1533034616681670 PMC5762029

[R19] Howard M, Beltran C, Mayo CS and Herman MG 2014 Effects of minimum monitor unit threshold on spot scanning proton plan quality Med. Phys 41 091703 25186378 10.1118/1.4892057

[R20] Laissue JA, Blattmann H and Slatkin DN 2012 Alban Köhler (1874–1947): erfinder der Gittertherapie Z. Med. Phys 22 90–99 21862299 10.1016/j.zemedi.2011.07.002

[R21] Li W, Lin Y, Li H, Rotondo R and Gao H 2023a An iterative convex relaxation method for proton LET optimization Phys. Med. Biol 68 055002 10.1088/1361-6560/acb88d PMC10037460 36731144

[R22] Li W, Zhang W, Lin Y, Chen RC and Gao H 2023b Fraction optimization for hybrid proton-photon treatment planning Med. Phys 50 3311–23 36786202 10.1002/mp.16297 PMC10271913

[R23] Lin Y, Clasie B, Liu T, McDonald M, Langen KM and Gao H 2019 Minimum-MU and sparse-energy-level (MMSEL) constrained inverse optimization method for efficiently deliverable PBS plans Phys. Med. Biol 64 205001 31530746 10.1088/1361-6560/ab4529

[R24] Lin Y, Kooy H, Craft D, Depauw N, Flanz J and Clasie B 2016 A Greedy reassignment algorithm for the PBS minimum monitor unit constraint Phys. Med. Biol 61 4665–78 27245098 10.1088/0031-9155/61/12/4665

[R25] Liu G, Li X, Qin A, Zhou J, Zheng W, Zhao L and Ding X 2021 Is proton beam therapy ready for single fraction spine SBRS?–a feasibility study to use spot-scanning proton arc (SPArc) therapy to improve the robustness and dosimetric plan quality Acta Oncol. 60 653–7 33645429 10.1080/0284186X.2021.1892183

[R26] Mayr NA 2024 Practice patterns of spatially fractionated radiation therapy: a clinical practice survey Adv. Radiat. Oncol 9 101308 38405319 10.1016/j.adro.2023.101308 PMC10885580

[R27] Mohiuddin M, Curtis DL, Grizos WT and Komarnicky L 1990 Palliative treatment of advanced cancer using multiple nonconfluent pencil beam radiation: a pilot study Cancer 66 114–8 1693874 10.1002/1097-0142(19900701)66:1<114::aid-cncr2820660121>3.0.co;2-l

[R28] Mohiuddin M, Lynch C, Gao M and Hartsell W 2020 Early clinical results of proton spatially fractionated GRID radiation therapy (SFGRT) Br. J. Radiol 93 20190572 31651185 10.1259/bjr.20190572 PMC7066961

[R29] Mossahebi S, Molitoris JK, Poirier Y, Jatczak J, Zhang B, Mohindra P, Yi B, Regine WF and Yi B 2024 Clinical implementation and dosimetric evaluation of a robust proton lattice planning strategy using primary and robust complementary beams Int. J. Radiat. Oncol. Biol. Phys 120 1149–58 38936634 10.1016/j.ijrobp.2024.06.009

[R30] Narayanasamy G, Zhang X, Meigooni A, Paudel N, Morrill S, Maraboyina S and Penagaricano J 2017 Therapeutic benefits in grid irradiation on Tomotherapy for bulky, radiation-resistant tumors Acta Oncol. 56 1043–7 28270018 10.1080/0284186X.2017.1299219

[R31] Nesterov Y 2018 Lectures on Convex Optimization (Springer)

[R32] Potez M, Fernandez-Palomo C, Bouchet A, Trappetti V, Donzelli M, Krisch M, Laissue J, Volarevic V and Djonov V 2019 Synchrotron microbeam radiation therapy as a new approach for the treatment of radioresistant melanoma: potential underlying mechanisms Int. J. Radiat. Oncol. Biol. Phys 105 1126–36 31461675 10.1016/j.ijrobp.2019.08.027

[R33] Prezado Y 2022 Divide and conquer: spatially fractionated radiation therapy Expert Rev. Mol. Med 24 e3

[R34] Shan J, An Y, Bues M, Schild SE and Liu W 2018 Robust optimization in IMPT using quadratic objective functions to account for the minimum MU constraint Med. Phys 45 460–9 29148570 10.1002/mp.12677 PMC5774242

[R35] Shen H, Zhang G, Lin Y, Rotondo RL, Long Y and Gao H 2023 Beam angle optimization for proton therapy via group-sparsity based angle generation method Med. Phys 50 3258–73 36965109 10.1002/mp.16392 PMC10272076

[R36] Tinganelli W and Durante M 2020 Carbon ion radiobiology Cancers 12 3022 33080914 10.3390/cancers12103022 PMC7603235

[R37] Unkelbach J 2018 Robust radiotherapy planning Phys. Med. Biol 63 22TR02 10.1088/1361-6560/aae659 30418942

[R38] Wieser HP, Cisternas E, Wahl N, Ulrich S, Stadler A, Mescher H and Bangert M 2017 Development of the open-source dose calculation and optimization toolkit matRad Med. Phys 44 2556–68 28370020 10.1002/mp.12251

[R39] Wu X, Ahmed MM, Wright J, Gupta S, Pollack A and Ahmed MM 2010 On modern technical approaches of three-dimensional high-dose lattice radiotherapy (LRT) Cureus 2

[R40] Wuyckens S, Saint-Guillain M, Janssens G, Zhao L, Li X, Ding X, Souris K, Lee JA and Souris K 2022 Treatment planning in arc proton therapy: comparison of several optimization problem statements and their corresponding solvers Comput. Biol. Med 148 105609 35803749 10.1016/j.compbiomed.2022.105609

[R41] Zhang G, Long Y, Lin Y, Chen RC and Gao H 2023a A treatment plan optimization method with direct minimization of number of energy jumps for proton arc therapy Phys. Med. Biol 68 085001 10.1088/1361-6560/acc4a7 PMC10112536 36921353

[R42] Zhang G, Shen H, Lin Y, Chen RC, Long Y and Gao H 2022 Energy layer optimization via energy matrix regularization for proton spot-scanning arc therapy Med. Phys 49 5752–62 35848227 10.1002/mp.15836 PMC9474698

[R43] Zhang H 2020 Photon GRID radiation therapy: a physics and dosimetry white paper from the radiosurgery society (RSS) GRID/LATTICE, microbeam and FLASH radiotherapy working group Radiat. Res 194 665–77 33348375 10.1667/RADE-20-00047.1

[R44] Zhang H and Mayr N 2023 Spatially Fractionated, Microbeam and Flash Radiation Therapy-A Physics and Multi-Disciplinary Approach (The IOP Publishing)

[R45] Zhang W, Li W, Lin Y, Wang F, Chen RC and Gao H 2023b TVL1-IMPT: optimization of peak-to-valley dose ratio via joint total-variation and l1 dose regularization for spatially fractionated pencil-beam-scanning proton therapy Int. J. Radiat. Oncol. Biol. Phys 115 768–78 36155212 10.1016/j.ijrobp.2022.09.064 PMC10155885

[R46] Zhang W, Lin Y, Wang F, Badkul R, Chen RC and Gao H 2023c Lattice position optimization for LATTICE therapy Med. Phys 50 7359–67 37357825 10.1002/mp.16572 PMC11058082

[R47] Zhang X, Penagaricano J, Yan Y, Liang X, Morrill S, Griffin RJ and Ratanatharathorn V 2016 Spatially fractionated radiotherapy (GRID) using helical tomotherapy J. Appl. Clin. Med. Phys 17 396–407 26894367 10.1120/jacmp.v17i1.5934 PMC5690194

[R48] Zhu XR, Sahoo N, Zhang X, Robertson D, Li H, Choi S, Gillin MT and Gillin MT 2010 Intensity modulated proton therapy treatment planning using single-field optimization: the impact of monitor unit constraints on plan quality Med. Phys 37 1210–9 20384258 10.1118/1.3314073 PMC2837732

[R49] Zhu YN, Zhang X, Lin Y, Lominska C and Gao H 2023 An orthogonal matching pursuit optimization method for solving minimum-monitor-unit problems: applications to proton IMPT, ARC and FLASH Med. Phys 50 4710–20 37427749 10.1002/mp.16577 PMC11031273

