# Supplementary material for: Proton ARC based LATTICE radiation therapy: feasibility study, energy layer optimization and LET optimization
Source: Phys Med Biol. Author manuscript; Available in PMC 2026 Mar 10. (PMC12973319; doi:10.1088/1361-6560/ad8855)
Supplement: Supplementary Data [file NIHMS2136628-supplement-Supplementary_Data.pdf]

Supplementary Material to

<u>Proton ARC based LATTICE radiation therapy: feasibility study, energy layer optimization and LET optimization</u>

The following dosimetric comparison follows the recommendation by the RSS SFRT working group [39].

Table S1. **PTV planning.** The plan-quality dosimetric quantities from left to right: $D_{x\%}$: dose covering x% of target. D_{mean}: mean target dose. EUD: equivalent uniform dose for target. gEUD: generalized EUD for stomach (abdomen) and esophagus (lung). The dose unit is Gy.

Cases		$D_{5\%}$	$D_{10\%}$	$D_{20\%}$	$D_{50\%}$	$D_{90\%}$	D_{mean}	$D_{90\%}$/ $D_{10\%}$	EUD	gEUD
Abdomen	IMPT	8.83	5.88	3.88	3.06	2.32	3.73	0.40	1.72	0.324
	ARC	7.11	4.58	3.07	2.63	2.13	3.17	0.46	2.45	0.251
	ELO	7.90	5.19	3.38	2.79	2.20	3.39	0.42	2.45	0.265
Lung	IMPT	10.3	6.75	4.20	3.03	2.28	3.89	0.33	1.97	0.496
	ARC	8.42	4.94	3.11	2.65	2.21	3.30	0.45	2.17	0.288
	ELO	9.48	5.93	3.51	2.86	2.23	3.59	0.38	2.17	0.348

Table S2. **CTV planning with robust optimization.** The plan-quality dosimetric quantities from left to right: $D_{x\%}$: dose covering x% of target. D_{mean}: mean target dose. EUD: equivalent uniform dose for target. gEUD: generalized EUD for stomach (abdomen) and esophagus (lung). The dose unit is Gy.

Cases		$D_{5\%}$	$D_{10\%}$	$D_{20\%}$	$D_{50\%}$	$D_{90\%}$	D_{mean}	$D_{90\%}$/ $D_{10\%}$	EUD	gEUD
Abdomen	IMPT	7.85	5.55	3.64	2.51	2.18	3.36	0.39	2.45	0.351
	ARC	7.22	5.16	3.58	2.41	2.10	3.13	0.41	2.45	0.289
	ELO	7.22	5.12	3.38	2.39	2.13	3.09	0.42	2.45	0.355
Lung	IMPT	10.1	7.15	4.53	2.80	2.23	3.82	0.31	1.97	0.441
	ARC	9.47	6.67	4.61	3.00	2.18	3.81	0.33	2.17	0.432
	ELO	9.58	6.64	4.06	2.64	2.21	3.58	0.33	2.17	0.205

Table S3. **Quality-efficiency multi-criteria optimization.** The plan-quality dosimetric quantities from left to right: $D_{x\%}$: dose covering x% target dose. D_{mean}: mean dose of the target. EUD: equivalent uniform dose for target. gEUD: generalized EUD for stomach. The dose unit is Gy.

Cases		$D_{5\%}$	$D_{10\%}$	$D_{20\%}$	$D_{50\%}$	$D_{90\%}$	D_{mean}	$D_{90\%}$/ $D_{10\%}$	EUD	gEUD
Abdomen	Case1	7.12	4.58	3.15	2.65	2.14	3.18	0.46	2.45	0.250
	Case2	7.90	5.19	3.38	2.79	2.20	3.39	0.42	2.45	0.265
	Case3	7.92	5.31	3.44	2.81	2.21	3.42	0.42	2.45	0.375

Table S4. **LET optimization.** The plan-quality dosimetric quantities from left to right: $D_{x\%}$: dose covering x% target dose. D_{mean}: mean dose of the target. EUD: equivalent uniform dose for target. gEUD: generalized EUD for esophagus. The dose unit is Gy.

Cases		$D_{5\%}$	$D_{10\%}$	$D_{20\%}$	$D_{50\%}$	$D_{90\%}$	D_{mean}	$D_{90\%}$/ $D_{10\%}$	EUD	gEUD
Lung	IMPT	6.85	4.48	2.79	2.01	1.51	2.58	0.22	1.77	0.354
	IMPT-LET1	6.85	4.49	2.81	2.04	1.57	2.61	0.23	1.77	0.349
	IMPT-LET2	6.90	4.59	2.86	2.00	1.45	2.58	0.21	1.77	0.371
	ARC	6.52	3.83	2.41	2.05	1.71	2.55	0.26	1.97	0.230
	ARC-LET1	6.55	3.98	2.56	2.09	1.80	2.62	0.28	1.97	0.222
	ARC-LET2	6.51	3.82	2.39	2.03	1.64	2.52	0.25	1.97	0.236

Table S5. **Protons v.s. photons.** The plan-quality dosimetric quantities from left to right: $D_{x\%}$: dose covering x% target dose. D_{mean}: mean dose of the target. EUD: equivalent uniform dose for target. gEUD: generalized EUD for esophagus. The dose unit is Gy.

Cases		$D_{5\%}$	$D_{10\%}$	$D_{20\%}$	$D_{50\%}$	$D_{90\%}$	D_{mean}	$D_{90\%}$/ $D_{10\%}$	EUD	gEUD
Lung	IMRT	14.4	10.7	7.59	4.28	2.60	5.72	0.24	1.97	0.883
	IMPT	10.3	6.75	4.20	3.03	2.28	3.89	0.34	1.97	0.496
	VMAT	8.89	6.20	4.13	2.67	2.21	3.58	0.36	2.17	0.653
	ARC	8.41	4.94	3.11	2.65	2.21	3.30	0.45	2.17	0.288

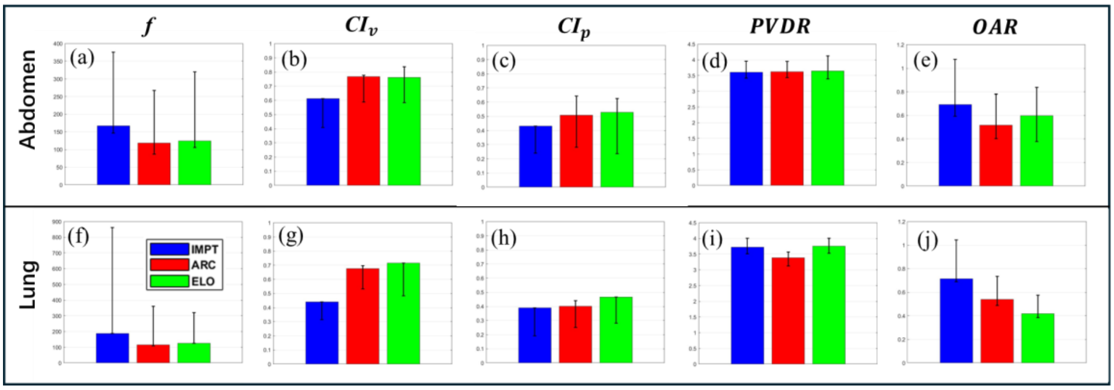

Figure S1. **Robust evaluation.** (a)-(e) Histogram of optimization objective value *f*, CI for valley dose CI_v, CI for peak dose CI_p, PVDR, and mean OAR dose D_{OAR} for abdomen using three different methods. (f)-(j) Histogram of optimization objective value *f*, CI for valley dose CI_v, CI for peak dose CI_p, PVDR, and mean OAR dose D_{OAR} for lung using three different methods.
